# Supplementary material for: Raspberry Ketone [4-(4-Hydroxyphenyl)-2-Butanone] Differentially Effects Meal Patterns and Cardiovascular Parameters in Mice
Source: Nutrients. 2020 Jun 11;12(6):1754. doi: 10.3390/nu12061754 (PMC7353175; doi:10.3390/nu12061754)
Supplement: Supplementary file 1 [file nutrients-12-01754-s001.pdf]

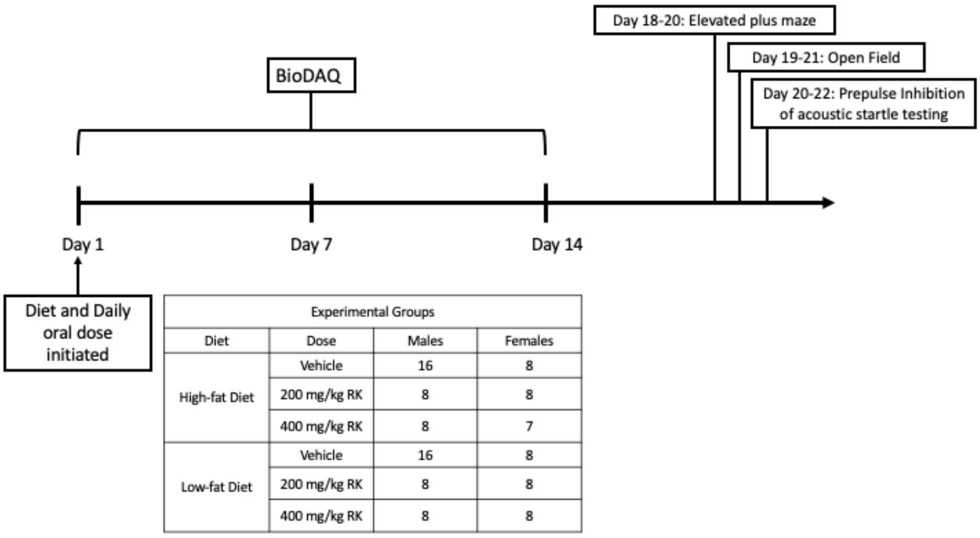

Experimental Groups			
Diet	Dose	Males	Females
High-fat Diet	Vehicle	16	8
	200 mg/kg RK	8	8
	400 mg/kg RK	8	7
Low-fat Diet	Vehicle	16	8
	200 mg/kg RK	8	8
	400 mg/kg RK	8	8

Figure S1. Experimental design for measurement of meal patterns and behavioral outcomes during exposure to respective diet and dose treatments.

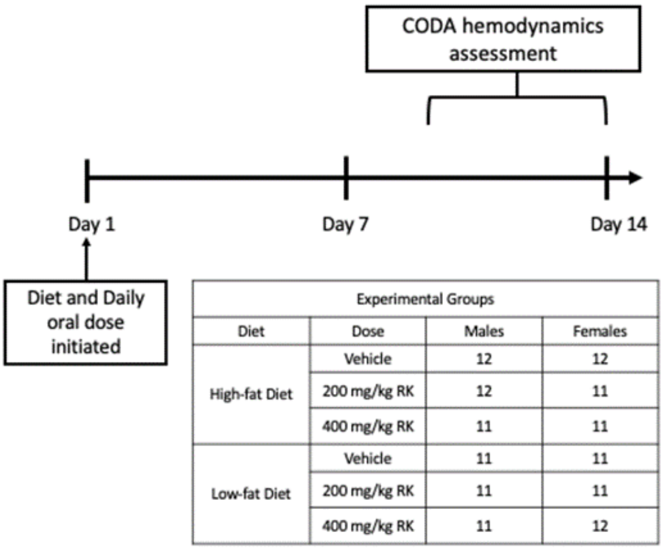

Experimental Groups			
Diet	Dose	Males	Females
High-fat Diet	Vehicle	12	12
	200 mg/kg RK	12	11
	400 mg/kg RK	11	11
Low-fat Diet	Vehicle	11	11
	200 mg/kg RK	11	11
	400 mg/kg RK	11	12

Figure S2. Experimental design for measurement of hemodynamic parameters after 14-day exposure to respective diet and dose treatments.

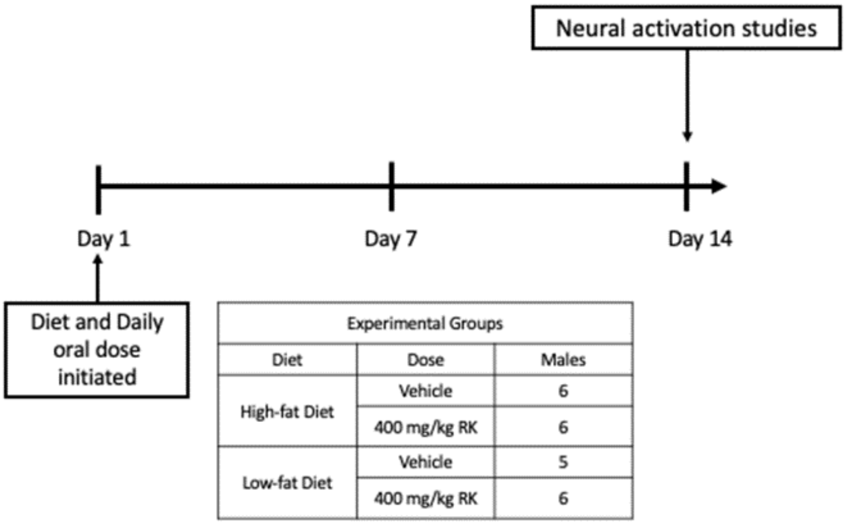

Experimental Groups		
Diet	Dose	Males
High-fat Diet	Vehicle	6
	400 mg/kg RK	6
Low-fat Diet	Vehicle	5
	400 mg/kg RK	6

Figure S3. Experimental design to study activation of caudal hindbrain in response to respective diet and dose treatments.
